# Nurses in the lead: a qualitative study on the development of distinct nursing roles in daily nursing practice

**DOI:** 10.1186/s12912-021-00613-3

**Published:** 2021-06-14

**Authors:** Jannine van Schothorst–van Roekel, Anne Marie J.W.M. Weggelaar-Jansen, Carina C.G.J.M. Hilders, Antoinette A. De Bont, Iris Wallenburg

**Affiliations:** Erasmus School of Health Policy & Management (ESHPM), Erasmus University, Rotterdam, The Netherlands

**Keywords:** Evidence-based practice, nursing practice, Policy, Registered nurses, Vocational-trained nurses, Role development, Role distinctions, Skill mix, Qualitative study

## Abstract

**Background:**

Transitions in healthcare delivery, such as the rapidly growing numbers of older people and increasing social and healthcare needs, combined with nursing shortages has sparked renewed interest in differentiations in nursing staff and skill mix. Policy attempts to implement new competency frameworks and job profiles often fails for not serving existing nursing practices. This study is aimed to understand how licensed vocational nurses (VNs) and nurses with a Bachelor of Science degree (BNs) shape distinct nursing roles in daily practice.

**Methods:**

A qualitative study was conducted in four wards (neurology, oncology, pneumatology and surgery) of a Dutch teaching hospital. Various ethnographic methods were used: shadowing nurses in daily practice (65h), observations and participation in relevant meetings (n=56), informal conversations (up to 15 h), 22 semi-structured interviews and member-checking with four focus groups (19 nurses in total). Data was analyzed using thematic analysis.

**Results:**

Hospital nurses developed new role distinctions in a series of small-change experiments, based on action and appraisal. Our findings show that: (1) this developmental approach incorporated the nurses’ invisible work; (2) nurses’ roles evolved through the accumulation of small changes that included embedding the new routines in organizational structures; (3) the experimental approach supported the professionalization of nurses, enabling them to translate national legislation into hospital policies and supporting the nurses’ (bottom-up) evolution of practices. The new roles required the special knowledge and skills of Bachelor-trained nurses to support healthcare quality improvement and connect the patients’ needs to organizational capacity.

**Conclusions:**

Conducting small-change experiments, anchored by *action and appraisal* rather than by *design*, clarified the distinctions between vocational and Bachelor-trained nurses. The process stimulated personal leadership and boosted the responsibility nurses feel for their own development and the nursing profession in general. This study indicates that experimental nursing role development provides opportunities for nursing professionalization and gives nurses, managers and policymakers the opportunity of a ‘two-way-window’ in nursing role development, aligning policy initiatives with daily nursing practices.

**Supplementary Information:**

The online version contains supplementary material available at 10.1186/s12912-021-00613-3.

## Background

The aging population and mounting social and healthcare needs are challenging both healthcare delivery and the financial sustainability of healthcare systems [1, 2]. Nurses play an important role in facing these contemporary challenges [3, 4]. However, nursing shortages increase the workload which, in turn, boosts resignation numbers of nurses [5, 6]. Research shows that nurses resign because they feel undervalued and have insufficient control over their professional practice and organization [7, 8]. This issue has sparked renewed interest in nursing role development [9–11]. A role can be defined by the activities assumed by one person, based on knowledge, modulated by professional norms, a legislative framework, the scope of practice and a social system [12, 9].

New nursing roles usually arise through task specialization [13, 14] and the development of advanced nursing roles [15, 16]. Increasing attention is drawn to role distinction within nursing teams by differentiating the staff and skill mix to meet the challenges of nursing shortages, quality of care and low job satisfaction [17, 18]. The staff and skill mix include the roles of enrolled nurses, registered nurses, and nurse assistants [19, 20]. Studies on differentiation in staff and skill mix reveal that several countries struggle with the composition of nursing teams [21–23].

Role distinctions between licensed vocational-trained nurses (VNs) and Bachelor of Science-trained nurses (BNs) has been heavily debated since the introduction of the higher nurse education in the early 1970s, not only in the Netherlands [24, 25] but also in Australia [26, 27], Singapore [20] and the United States of America [28, 29]. Current debates have focused on the difficulty of designing distinct nursing roles. For example, Gardner et al., revealed that registered nursing roles are not well defined and that job profiles focus on direct patient care [30]. Even when distinct nursing roles are described, there are no proper guidelines on how these roles should be differentiated and integrated into daily practice. Although the value of differentiating nursing roles has been recognized, it is still not clear how this should be done or how new nursing roles should be embedded in daily nursing practice. Furthermore, the consequences of these roles on nursing work has been insufficiently investigated [31].

This study reports on a study of nursing teams developing new roles in daily nursing hospital practice. In 2010, the Dutch Ministry of Health announced a law amendment (the Individual Health Care Professions Act) to formalize the distinction between VNs and BNs. The law amendment made a distinction in responsibilities regarding complexity of care, coordination of care, and quality improvement. Professional roles are usually developed top-down at policy level, through competency frameworks and job profiles that are subsequently implemented in nursing practice. In the Dutch case, a national expert committee made two distinct job profiles [32]. Instead of prescribing role implementation, however, healthcare organizations were granted the opportunity to develop these new nursing roles in practice, aiming for a more practice-based approach to reforming the nursing workforce. This study investigates a Dutch teaching hospital that used an experimental development process in which the nurses developed role distinctions by ‘doing and appraising’. This iterative process evolved in small changes [33–36], based on nurses’ thorough knowledge of professional practices [37] and leadership role [38–40].

According to Abbott, the constitution of a new role is a competitive action, as it always leads to negotiation of new openings for one profession and/or degradation of adjacent professions [41]. Additionally, role differentiation requires negotiation between different professionals, which always takes place in the background of historical professionalization processes and vested interests resulting in power-related issues [42–44]. Recent studies have described the differentiation of nursing roles to other professionals, such as nurse practitioners and nurse assistants, but have focused on evaluating shifts in nursing tasks and roles [31]. Limited research has been conducted on differentiating between the different roles of registered nurses and the involvement of nurses themselves in developing new nursing roles. An ethnographic study was conducted to shed light on the nurses’ work of seeking openings and negotiating roles and responsibilities and the consequences of role distinctions, against a background of historically shaped relationships and patterns.

## Methods

### Aim

The study aimed to understand the formulation of nursing role distinctions between different educational levels in a development process involving experimental action (doing) and appraisal.

### Design

We conducted an ethnographic case study. This design was commonly used in nursing studies in researching changing professional practices [45, 46]. The researchers gained detailed insights into the nurses’ actions and into the finetuning of their new roles in daily practice, including the meanings, beliefs and values nurses give to their roles [47, 48]. This study complied with the consolidated criteria for reporting qualitative research (COREQ) checklist.

### Setting and participants

Our study took place in a purposefully selected Dutch teaching hospital (481 beds, 2,600 employees including 800 nurses). Historically, nurses in Dutch hospitals have vocational training. The introduction of higher nursing education in 1972 prompted debates about distinguishing between vocational-trained nurses (VNs) and bachelor-trained nurses (BNs). For a long time, VNs resisted a role distinction, arguing that their work experience rendered them equally capable to take care of patients and deal with complex needs. As a result, VNs and BNs carry out the same duties and bear equal responsibility. To experiment with role distinctions in daily practice, the hospital management and project team selected a convenience but representative sample of wards. Two general (neurology and surgery) and two specific care (oncology and pneumatology) wards were selected as they represent the different compositions of nursing educational levels (VN, BN and additional specialized training). The demographic profile for the nursing teams is shown in Table 1. The project team, comprising nursing policy staff, coaches and HR staff (*N* = 7), supported the four (nursing) teams of the wards in their experimental development process (131 nurses; 32 % BNs and 68 % VNs, including seven senior nurses with an organizational role). We also studied the interactions between nurses and team managers (*N* = 4), and the CEO (*N* = 1) in the meetings.

**Table 1:** Demographics of the study participants^a^

Variables	Numbers
Wards	
Oncology	22
Neurology	19
Surgery	15
Pulmonology	16
Age	
younger than 25	14
25 till 34	28
35 till 44	10
45 till 54	15
55+	5
Average	35
Gender	
male	4
female	68
Education level	
VN	46
BN	26
Current role	
VN	44
BN	25
Senior	3
Work hours	
<28	11
≥28	61
Years of experience	
<3 years	23
≥3 and < 5 years	9
≥ 5 and < 10 years	11
≥ 10 years	29

^a^Demographic data derived from a quantitative study that was conducted in parallel on the same sample (72 respondents, 55% of the study participants, representative for the total sample)

### Data collection

Data was collected between July 2017 and January 2019. A broad selection of respondents was made based on the different roles they performed. Respondents were personally approached by the first author, after close consultation with the team managers. Four qualitative research methods were used iteratively combining collection and analysis, as is common in ethnographic studies [45] (see Table 2).

1. Shadowing nurses (i.e. observations and questioning nurses about their work) on shift (65 h in total) was conducted to observe behavior in detail in the nurses’ organizational and social setting [49, 50], both in existing practices and in the messy fragmented process of developing distinct nursing roles. The notes taken during shadowing were worked up in thick descriptions [46].
2. Observation and participation in four types of meetings. The first and second authors attended: (1) kick-off meetings for the nursing teams (*n* = 2); (2) bi-monthly meetings (*n* = 10) between BNs and the project team to share experiences and reflect on the challenges, successes and failures; and (3) project group meetings at which the nursing role developmental processes was discussed (*n* = 20). Additionally, the first author observed nurses in ward meetings discussing the nursing role distinctions in daily practice (*n* = 15). Minutes and detailed notes also produced thick descriptions [51]. This fieldwork provided a clear understanding of the experimental development process and how the respondents made sense of the challenges/problems, the chosen solutions and the changes to their work routines and organizational structures. During the fieldwork, informal conversations took place with nurses, nursing managers, project group members and the CEO (app. 15 h), which enabled us to reflect on the daily experiences and thus gain in-depth insights into practices and their meanings. The notes taken during the conversations were also written up in the thick description reports, shortly after, to ensure data validity [52]. These were completed with organizational documents, such as policy documents, activity plans, communication bulletins, formal minutes and in-house presentations.
3. Semi-structured interviews lasting 60–90 min were held by the first author with 22 respondents: the CEO (*n* = 1), middle managers (*n* = 4), VNs (*n* = 6), BNs (*n* = 9, including four senior nurses), paramedics (*n* = 2) using a predefined topic list based on the shadowing, observations and informal conversations findings. In the interviews, questions were asked about task distinctions, different stakeholder roles (i.e., nurses, managers, project group), experimental approach, and added value of the different roles and how they influence other roles. General open questions were asked, including: “How do you distinguish between tasks in daily practice?”. As the conversation proceeded, the researcher asked more specific questions about what role differentiation meant to the respondent and their opinions and feelings. For example: “what does differentiation mean for you as a professional?”, and “what does it mean for you daily work?”, and “what does role distinction mean for collaboration in your team?” The interviews were tape-recorded (with permission), transcribed verbatim and anonymized.
4. The fieldwork period ended with four focus groups held by the first author on each of the four nursing wards (*N* = 19 nurses in total: nine BNs, eight VNs, and two senior nurses). The groups discussed the findings, such as (nurses’ perceptions on) the emergence of role distinctions, the consequences of these role distinctions for nursing, experimenting as a strategy, the elements of a supportive environment and leadership. Questions were discussed like: “which distinctions are made between VN and BN roles?”, and “what does it mean for VNs, BNs and senior nurses?”. During these meetings, statements were also used to provoke opinions and discussion, e.g., “The role of the manager in developing distinct nursing roles is…”. With permission, all focus groups were audio recorded and the recordings were transcribed verbatim. The focus groups also served for member-checking and enriched data collection, together with the reflection meetings, in which the researchers reflected with the leader and a member of the project group members on program, progress, roles of actors and project outcomes. Finally, the researchers shared a report of the findings with all participants to check the credibility of the analysis.

**Table 2:** Data collection methods for both cases, excluding document study

Hospital wards	Participants	Shadowing nurses	Conversations	Interviews	Meetings
*neurology *surgery *oncology *pulmonology	Ward nurses: VNs, BSNs, Senior nurses (*n* = 131) Managers (*n* = 4) Project group (*n* = 7) Top manager (*n* = 1)	65 h approx.	15 h approx.	Top manager (*n* = 1), Nurse managers (*n* = 4) VNs (*n* = 6) BSNs (*n* = 9) Paramedics (*n* = 2) Total interviews *n* = 22 60–90 min each	Kick-off meeting: nursing team, manager, project group members (*n* = 2) Ward meetings: BSNs, VNs, senior nurses, manager (*n* = 15) Interdepartmental meetings: 2 nurses per team, team managers, project group members (*n* = 10) Project group meetings: nurse project leader, nurse project member, teachers/ coaches, HR staff, researchers (*n* = 20) Team focus groups (*n* = 4; 19 nurses in total) Reflection meetings: project leader and member of the Nurse Advisory dept, 2 researchers (*n* = 9) Total meetings *n* = 60

### Data analysis

Data collection and inductive thematic analysis took place iteratively [45, 53]. The first author coded the data (i.e. observation reports, interview and focus group transcripts), basing the codes on the research question and theoretical notions on nursing role development and distinctions. In the next step, the research team discussed the codes until consensus was reached. Next, the first author did the thematic coding, based on actions and interactions in the nursing teams, the organizational consequences of their experimental development process, and relevant opinions that steered the development of nurse role distinctions (see Additional file). Iteratively, the research team developed preliminary findings, which were fed back to the respondents to validate our analysis and deepen our insights [54]. After the analysis of the additional data gained in these validating discussions, codes were organized and re-organized until we had a coherent view.

### Rigor

Ethnography acknowledges the influence of the researcher, whose own (expert) knowledge, beliefs and values form part of the research process [48]. The first author was involved in the teams and meetings as an observer-as-participant, to gain in-depth insight, but remained research-oriented [55]. The focus was on the study of nursing actions, routines and accounts, asking questions to obtain insights into underlying assumptions, which the whole research group discussed to prevent ‘going native’ [56, 57]. Rigor was further ensured by triangulating the various data resources (i.e. participants and research methods), purposefully gathered over time to secure consistency of findings and until saturation on a specific topic was reached [54]. The meetings in which the researchers shared the preliminary findings enabled nurses to make explicit their understanding of what works and why, how they perceived the nursing role distinctions and their views on experimental development processes.

### Ethical considerations

All participants received verbal and written information, ensuring that they understood the study goals and role of the researcher [48]. Participants were informed about their voluntary participation and their right to end their contribution to the study. All gave informed consent. The study was performed in accordance with the Declaration of Helsinki and was approved by the Erasmus Medical Ethical Assessment Committee in Rotterdam (MEC-2019-0215), which also assessed the compliance with GDPR.

## Results

Our findings reveal how nurses gradually shaped new nursing role distinctions in an experimental process of action and appraisal and how the new BN nursing roles became embedded in new nursing routines, organizational routines and structures. Three empirical appeared from the systematic coding: (1) distinction based on complexity of care; (2) organizing hospital care; and (3) evidence-based practices (EBP) in quality improvement work.

### Distinction based on complexity of care

Initially, nurses distinguished the VN and BN roles based on the complexity of patient care, as stated in national job profiles [32]. BNs were supposed to take care of clinically complex patients, rather than VNs, although both VNs and BNs had been equally taking care of every patient category. To distinguish between highly and less complex patient care, nurses developed a complexity measurement tool. This tool enabled classification of the predictability of care, patient’s degree of self-reliance, care intensity, technical nursing procedures and involvement of other disciplines. However, in practice, BNs questioned the validity of assessing a patient’s care complexity, because the assessments of different nurses often led to different outcomes. Furthermore, allocating complex patient care to BNs impacted negatively on the nurses’ job satisfaction, organizational routines and ultimately the quality of care. VNs experienced the shift of complex patient care to BNs as a diminution of their professional expertise. They continuously stressed their competencies and questioned the assigned levels of complexity, aiming to prevent losses to their professional tasks:

‘Now we’re only allowed to take care of COPD patients and people with pneumonia, so no more young boys with a pneumothorax drain. Suddenly we are not allowed to do that. (…) So, your [professional] world is getting smaller. We don’t like that at all. So, we said: We used to be competent, so why aren’t we anymore?’ (Interview VN1, in-service trained nurse).

In discussing complexity of care, both VNs and BNs (re)discovered the competencies VNs possess in providing complex daily care. BNs acknowledged the contestability of the distinction between VN and BN roles related to patient care complexity, as the next quote shows:

‘Complexity, they always make such a fuss about it. (…) At a given moment you’re an expert in just one certain area; try then to stand out on your ward. (…) When I go to GE [gastroenterology] I think how complex care is in here! (…) But it’s also the other way around, when I’m the expert and know what to expect after an angioplasty, or a bypass, or a laparoscopic cholecystectomy (…) When I’ve mastered it, then I no longer think it’s complex, because I know what to expect!’ (Interview BN1, 19-07-2017).

This quote illustrates how complexity was shaped through clinical experience. What complex care *is*, is influenced by the years of doing nursing work and hence is individual and remains invisible. It is not formally valued [58] because it is not included in the BN-VN competency model. This caused dissatisfaction and feelings of demotion among VNs. The distinction in complexities of care was also problematic for BNs. Following the complexity tool, recently graduated BNs were supposed to look after highly complex patients. However, they often felt insecure and needed the support of more experienced (VN) colleagues – which the VNs perceived as a recognition of their added value and evidence of the failure of the complexity tool to guide division of tasks. Also, mundane issues like holidays, sickness or pregnancy leave further complicated the use of the complexity tool as a way of allocating patients, as it decreased flexibility in taking over and swapping shifts, causing dissatisfaction with the work schedule and leading to problems in the continuity of care during evening, night and weekend shifts. Hence, the complexity tool disturbed the flexibility in organizing the ward and held possible consequences for the quality and safety of care (e.g. inexperienced BNs providing complex care), Ultimately, the complexity tool upset traditional teamwork, in which nurses more implicitly complemented each other’s competencies and ability to ‘get the work done’ [59]. As a result, role distinction based on ‘quantifiable’ complexity of care was abolished. Attention shifted to the development of an organizational and quality-enhancing role, seeking to highlight the added value of BNs – which we will elaborate on in the next section.

### Organizing hospital care

Nurses increasingly fulfill a coordinating role in healthcare, making connections across occupational, departmental and organizational boundaries, and ‘mediating’ individual patient needs, which Allen describes as organizing work [49]. Attempting to make a valuable distinction between nursing roles, BNs adopted coordinating management tasks at the ward level, taking over this task from senior nurses and team managers. BNs sought to connect the coordinating management tasks with their clinical role and expertise. An example is bed management, which involves comparing a ward’s bed capacity with nursing staff capacity [1, 60]. At first, BNs accompanied middle managers to the hospital bed review meeting to discuss and assess patient transfers. On the wards where this coordination task used to be assigned to senior nurses, the process of transferring this task to BNs was complicated. Senior nurses were reluctant to hand over coordinating tasks as this might undermine their position in the near future. Initially, BNs were hesitant to take over this task, but found a strategy to overcome their uncertainty. This is reflected in the next excerpt from fieldnotes:

Senior nurse: ‘First we have to figure out if it will work, don’t we? I mean, all three of us [middle manager, senior nurse, BN] can’t just turn up at the bed review meeting, can we? The BN has to know what to do first, otherwise she won’t be able to coordinate properly. We can’t just do it.’ BN: ‘I think we should keep things small, just start doing it, step by step. (…) If we don’t try it out, we don’t know if it works.’ (Field notes, 24-05-2018).

This excerpt shows that nurses gradually developed new roles as a series of matching tasks. Trying out and evaluating each step of development in the process overcame the uncertainty and discomfort all parties held [61]. Moreover, carrying out the new tasks made the role distinctions become apparent. The coordinating role in bed management, for instance, became increasingly embedded in the new BN nursing role. Experimenting with coordination allowed BNs prove their added value [62] and contributed to overall hospital performance as it combined daily working routines with their ability to manage bed occupancy, patient flow, staffing issues and workload. This was not an easy task. The next quote shows the complexity of creating room for this organizing role:

The BNs decide to let the VNs help coordinate the daily care, as some VNs want to do this task. One BN explains: ‘It’s very hard to say, you’re not allowed.’ The middle manager looks surprised and says that daily coordination is a chance to draw a clear distinction and further shape the role of BNs. The project group leader replies: ‘Being a BN means that you dare to make a difference [in distinctive roles]. We’re all newbies in this field, but we can use our shared knowledge. You can derive support from this task for your new role.’ (Field notes, 09-01-2018).

This excerpt reveals the BNs’ thinking on crafting their organizational role, turning down the VNs wishes to bear equal responsibility for coordinating tasks. Taking up this role touched on nurse identity as BNs had to overcome the delicate issue of equity [63], which has long been a core element of the Dutch nursing profession. Taking over an organization role caused discomfort among BNs, but at the same time provided legitimation for a role distinction.

Legitimation for this task was also gained from *external* sources, as the law amendment and the expert committee’s job descriptions both mentioned coordinating tasks. However, taking over coordinating tasks and having an organizing role in hospital care was not done as an ‘implementation’; rather it required a process of actively crafting and carving out this new role. We observed BNs choosing not to disclose that they were experimenting with taking over the coordinating tasks as they anticipated a lack of support from VNs:

BN: ‘We shouldn’t tell the VNs everything. We just need this time to give shape to our new role. And we all know who [of the colleagues] won’t agree with it. In my opinion, we’d be better off hinting at it at lunchtime, for example, to figure out what colleagues think about it. And then go on as usual.’ (Field notes, 12-06-2018).

BNs stayed ‘under the radar’, not talking explicitly about their fragile new role to protect the small coordination tasks they had already gained. By deliberately keeping the evaluation of their new task to themselves, they protected the transition they had set into motion. Thus, nurses collected small changes in their daily routines, developing a new role distinction step by step. Changes to single tasks accumulated in a new role distinction between BNs, VNs and senior nurses, and gave BNs a more hybrid nursing management role.

### Evidence-based practices in quality improvement work

Quality improvement appeared to be another key concern in the development of the new BN role. Quality improvement work used to be carried out by groups of senior nurses, middle managers and quality advisory staff. Not involved in daily routines, the working group focused on nursing procedures (e.g. changing infusion system and wound treatment protocols). In taking on this new role BNs tried different ways of incorporating EBP in their routines, an aspect that had long been neglected in the Netherlands. As a first step, BNs rearranged the routines of the working group. For example, a team of BNs conducted a quality improvement investigation of a patient’s formal’s complaint:

Twenty-two patients registered a pain score of seven or higher and were still discharged. The question for BNs was: how and why did this bad care happen? The BNs used electronic patient record to study data on the relations between pain, medication and treatment. Their investigation concluded: nurses do not always follow the protocols for high pain scores. Their improvement plan covered standard medication policy, clinical lessons on pain management and revisions to the patient information folder. One BN said: ‘I really loved investigating this improvement.’ (Field notes, 28-05-2018).

This fieldnote shows the joy quality improvement work can bring. During interviews, nurses said that it had given them a better grip on the outcome of nursing work. BNs felt the need to enhance their quality improvement tasks with their EBP skills, e.g. using clinical reasoning in bedside teaching, formulating and answering research questions in clinical lessons and in multi-disciplinary patient rounds to render nursing work more evidence based. The BNs blended EBP-related education into shift handovers and ward meetings, to show VNs the value of doing EBP [64]. In doing so, they integrated and fostered an EBP infrastructure of care provision, reflecting a new sense of professionalism and responsibility for quality of care.

However, learning how to blend EPB quality work in daily routines – ‘learning in practice’ –requires attention and steering. Although the BNs had a Bachelor’s degree, they had no experience of a quality-enhancing role in hospital practice [65]. In our case, the interplay between team members’ previous education and experienced shortcomings in knowledge and skills uncovered the need for further EBP training. This training established the BNs’ role as quality improvers in daily work and at the same time supported the further professionalization of both BNs and VNs. Although introducing the EBP approach was initially restricted to the BNs, it was soon realized that VNs should be involved as well, as nursing is a collaborative endeavor [1], as one team member (the trainer) put it:

‘I think that collaboration between BNs and VNs would add lots of value, because both add something different to quality work. I’d suggest that BNs could introduce the process-oriented, theoretical scope, while VNs could maybe focus on the patients’ interest.’ (Fieldnote, informal conversation, 11-06-2018).

During reflection sessions on the ward level and in the project team meetings BNs, informed by their previous experience with the complexity tool, revealed that they found it a struggle to do justice to everyone’s competencies. They wanted to use everyone’s expertise to improve the quality of patient care. They were for VNs being involved in the quality work, e.g. in preparing a clinical lesson, conducting small surveys, asking VNs to pose EBP questions and encourage VNs to write down their thoughts on flip over charts as means of engaging all team members.

These findings show that applying EPB in quality improvement is a relational practice driven by mutual recognition of one another’s competencies. This relational practice blended the BNs’ theoretical competence in EBP [66] with the VNs’ practical approach to the improvement work they did together. As a result, the blend enhanced the quality of daily nursing work and thus improved the quality of patient care and the further professionalization of the whole nursing team.

## Discussion

This study aimed to understand how an experimental approach enables differently educated nurses to develop new, distinct professional roles. Our findings show that roles cannot be distinguished by complexity of care; VNs and BNs are both able to provide care to patients with complex healthcare needs based on their knowledge and experience. However, role distinctions can be made on organizing care and quality improvement. BNs have an important role organizing care, for example arranging the patient flow on and across wards at bed management meetings, while VNs contribute more to organizing at the individual patient level. BNs play a key role in starting and steering quality improvement work, especially blending EBP in with daily nursing tasks, while VNs are involved but not in the lead. Working together on quality improvement boosts nursing professionalization and team development.

Our findings also show that the role development process is greatly supported by a series of small-change experiments, based on action and appraisal. This experimental approach supported role development in three ways. First, it incorporates both formal tasks and the invisible, unconscious elements of nursing work [49]. Usually, invisible work gets no formal recognition, for example in policy documents [55], whereas it is crucial in daily routines and organizational structures [49, 60]. Second, experimenting triggers an accumulation of small changes [33, 35] leading to the embeddedness of role distinctions in new nursing routines, allowing nurses to influence the organization of care. This finding confirms the observations of Reay et al. that nurses can create small changes in daily activities to craft a new nursing role, based on their thorough knowledge of their own practice and that of the other involved professional groups [37]. Although these changes are accompanied by tension and uncertainty, the process of developing roles generates a certain joy. Third, experimenting stimulated nursing professionalization, enabling the nurses to translate national legislation into hospital policy and supporting the nurses’ own (bottom-up) evolution of practices. Historically, nursing professionalization is strongly influenced by gender and education level [43] resulting in a subordinate position, power inequity and lack of autonomy [44]. Giving nurses the lead in developing distinct roles enables them to ‘engage in acts of power’ and obtain more control over their work. Fourth, experimenting contributes to role definition and clarification. In line with Poitras et al. [12] we showed that identifying and differentiating daily nursing tasks led to the development of two distinct and complementary roles. We have also shown that the knowledge base of roles and tasks includes both previous and additional education, as well as nursing experience.

Our study contributes to the literature on the development of distinct nursing roles [9–11] by showing that delineating new roles in formal job descriptions is not enough. Evidence shows that this formal distinction led particularly to the non-recognition, non-use and degradation [41] of VN competencies and discomforted recently graduated BNs. The workplace-based experimental approach in the hospital includes negotiation between professionals, the adoption process of distinct roles and the way nurses handle formal policy boundaries stipulated by legislation, national job profiles, and hospital documents, leading to clear role distinctions. In addition to Hughes [42] and Abbott [67] who showed that the delineation of formal work boundaries does not fit the blurred professional practices or individual differences in the profession, we show how the experimental approach leads to the clarification and shape of distinct professional practices.

Thus, an important implication of our study is that the professionals concerned should be given a key role in creating change [37, 39, 40]. Adding to Mannix et al. [38], our study showed that BNs fulfill a leadership role, which allows them to build on their professional role and identity. Through the experiments, BNs and VNs filled the gap between what they had learned in formal education, and what they do in daily practice [64, 65]. Experimenting integrates learning, appraising and doing much like going on ‘a journey with no fixed routes’ [34, 68] and no fixed job description, resulting in the enlargement of their roles.

Our study suggests that role development should involve professionalization at different educational levels, highlighting and valuing specific roles rather than distinguishing higher and lower level skills and competencies. Further research is needed to investigate what experimenting can yield for nurses trained at different educational levels in the context of changing healthcare practices, and which interventions (e.g., in process planning, leadership, or ownership) are needed to keep the development of nursing roles moving ahead. Furthermore, more attention should be paid to how role distinction and role differentiation influence nurse capacity, quality of care (e.g., patient-centered care and patient satisfaction), and nurses’ job satisfaction.

### Limitations

Our study was conducted on four wards of one teaching hospital in the Netherlands. This might limit the potential of generalizing our findings to other contexts. However, the ethnographic nature of our study gave us unique understanding and in-depth knowledge of nurses’ role development and distinctions, both of which have broader relevance. As always in ethnographic studies, the chances of ‘going native’ were apparent, and we tried to prevent this with ongoing reflection in the research team. Also, the interpretation of research findings within the Dutch context of nurse professionalization contributed to a more in-depth understanding of how nursing roles develop, as well as the importance of involving nurses themselves in the development of these roles to foster and support professional development.

We focused on role distinctions between VNs and BNs and paid less attention to (the collaboration with) other professionals or management. Further research is needed to investigate how nursing role development takes place in a broader professional and managerial constellation and what the consequences are on role development and healthcare delivery.

## Conclusions

This paper described how nurses crafted and shaped new roles with an experimental process. It revealed the implications of developing a distinct VN role and the possibility to enhance the BN role in coordination tasks and in steering and supporting EBP quality improvement work. Embedding the new roles in daily practice occurred through an accumulation of small changes. Anchored by *action and appraisal* rather than by *design*, the changes fostered by experiments have led to a distinction between BNs and VNs in the Netherlands. Furthermore, experimenting with nursing role development has also fostered the professionalization of nurses, encouraging nurses to translate knowledge into practice, educating the team and stimulating collaborative quality improvement activities.

This paper addressed the enduring challenge of developing distinct nursing roles at both the vocational and Bachelor’s educational level. It shows the importance of experimental nursing role development as it provides opportunities for the professionalization of nurses at different educational levels, valuing specific roles and tasks rather than distinguishing between higher and lower levels of skills and competencies. Besides, nurses, managers and policymakers can embrace the opportunity of a ‘two-way window’ in (nursing) role development, whereby distinct roles are outlined in general at policy levels, and finetuned in daily practice in a process of small experiments to determine the best way to collaborate in diverse contexts.

## Supplementary Information

**Additional file 1.**

## Data Availability

The data generated and analyzed during the current study is not publicly available to ensure data confidentiality but is available from the corresponding author on reasonable request and with the consent of the research participants.

## Abbreviations

BN: Bachelor-trained nurse
VN: Vocational-trained nurse
EBP: Evidence-based Practices
