# Supplementary material for: Nurses in the lead: a qualitative study on the development of distinct nursing roles in daily nursing practice
Source: BMC Nurs. 2021 Jun 14;20:97. doi: 10.1186/s12912-021-00613-3 (PMC8201810; doi:10.1186/s12912-021-00613-3)
Supplement: Supplementary file 1 — Additional file 1. [file 12912_2021_613_MOESM1_ESM.docx]

Themes on the development of distinct nursing roles

Subthemes

Codes

Using job profiles to distinguish roles

Distinguishing roles based on complexity of care

Taking clinical complexity and clinical experience into account

Nurses experiencing organizational complexities in differentiating nursing roles

Feelings of dissatisfaction and demotion among VNs

Making visible and negotiating the added value of VNs

Disturbance of flexibility in organizing nursing care

Nurses, managers

Distinct roles

Competencies

Education, educational levels

Job profiles

Complexity of care

Collaboration

Coordination

Negotiation

Legitimacy

Organization of care

Organizational routines

Quality of care

Experimental approach

Try outs

Evaluation

Related to:

Nursing role development

Invisible work

Organizing work

Organizational routines

Experimental approach

Distinction based on complexity of care

BNs adopt coordinating tasks

Connecting coordinating tasks with clinical roles and expertise

Enlarging existing nursing roles and routines

Accumulation of small steps in the development of a new role

Experiencing feelings of uncertainty among BNs in taking over coordinating tasks

BNs experiencing discomfort in creating differences between nurses

BNs proving their added value

Deriving legitimization from external sources

BNs staying under the radar to protect new roles

Organizing hospital care

Connecting EPB to daily work

Incorporating EBP in nursing routines

BNs experience the need to extend their QI knowledge and skills

Integrating learning and doing

VNs’ involvement in QI

Nursing role development as relational and remaining teamwork

Evidence-based practices in quality improvement work

Coding structure
